# Levosimendan for patients with severely reduced left ventricular systolic function and/or low cardiac output syndrome undergoing cardiac surgery: a systematic review and meta-analysis

**DOI:** 10.1186/s13054-017-1849-0

**Published:** 2017-10-19

**Authors:** Filippo Sanfilippo, Joshua B. Knight, Sabino Scolletta, Cristina Santonocito, Federico Pastore, Ferdinando L. Lorini, Luigi Tritapepe, Andrea Morelli, Antonio Arcadipane

**Affiliations:** 1 Department of Anesthesia and Intensive Care, Istituto Mediterraneo per i Trapianti e Terapie ad alta specializzazione (IRCCS-ISMETT), Via Tricomi 5, 90127 Palermo, Italy; 2 Department of Anesthesiology, University of Pittsburgh Medical Center, Pittsburgh, PA USA; 3 Unit of Anesthesia and Critical Care Medicine, Department of Medical Biotechnologies, Azienda Ospedaliera Universitaria Senese, Siena, Italy; 4 Department of Anaesthesia and Intensive Care, Papa Giovanni XXIII Hospital, Bergamo, Italy; 5 Department of Cardiovascular, Respiratory, Nephrological, Anaesthetic and Geriatric Sciences, Sapienza University of Rome, Rome, Italy; 6 Department of Anesthesiology and Intensive Care, Policlinico Umberto 1, Sapienza University of Rome, Rome, Italy

**Keywords:** Ejection fraction, Renal replacement therapy, Mortality, Intensive care

## Abstract

**Background:**

Previous studies have shown beneficial effects of levosimendan in high-risk patients undergoing cardiac surgery. Two large randomized controlled trials (RCTs), however, showed no advantages of levosimendan.

**Methods:**

We performed a systematic review and meta-analysis (MEDLINE and Embase from inception until March 30, 2017), investigating whether levosimendan offers advantages compared with placebo in high-risk cardiac surgery patients, as defined by preoperative left ventricular ejection fraction (LVEF) ≤ 35% and/or low cardiac output syndrome (LCOS). The primary outcomes were mortality at longest follow-up and need for postoperative renal replacement therapy (RRT). Secondary postoperative outcomes investigated included myocardial injury, supraventricular arrhythmias, development of LCOS, acute kidney injury (AKI), duration of mechanical ventilation, intensive care unit and hospital lengths of stay, and incidence of hypotension during drug infusion.

**Results:**

Six RCTs were included in the meta-analysis, five of which investigated only patients with LVEF ≤ 35% and one of which included predominantly patients with LCOS. Mortality was similar overall (OR 0.64 [0.37, 1.11], *p* = 0.11) but lower in the subgroup with LVEF < 35% (OR 0.51 [0.32, 0.82], *p* = 0.005). Need for RRT was reduced by levosimendan both overall (OR 0.63 [0.42, 0.94], *p* = 0.02) and in patients with LVEF < 35% (OR 0.55 [0.31, 0.97], *p* = 0.04). Among secondary outcomes, we found lower postoperative LCOS in patients with LVEF < 35% receiving levosimendan (OR 0.49 [0.27, 0.89], *p* = 0.02), lower overall AKI (OR 0.62 [0.42, 0.92], *p* = 0.02), and a trend toward lower mechanical support, both overall (*p* = 0.07) and in patients with LVEF < 35% (*p* = 0.05).

**Conclusions:**

Levosimendan reduces mortality in patients with preoperative severely reduced LVEF but does not affect overall mortality. Levosimendan reduces the need for RRT after high-risk cardiac surgery.

**Electronic supplementary material:**

The online version of this article (doi:10.1186/s13054-017-1849-0) contains supplementary material, which is available to authorized users.

## Background

Over the last two decades, the risk profile of patients undergoing cardiac surgery has increased significantly [1, 2], and currently more than 1 million cardiac surgery procedures are performed annually in the United States and Europe [3]. Older patients and those with a higher degree of comorbidities are currently referred for cardiac surgery [4], and, even if such patients may benefit from cardiac surgery, they are at increased risk for perioperative complications that result in high morbidity and mortality [5]. Patients with a severely depressed left ventricular ejection fraction (LVEF < 35%) are particularly at increased risk of developing postoperative low cardiac output syndrome (LCOS) [6], which in turn is associated with higher mortality [7].

Levosimendan, a calcium-sensitizing inotrope and an ATP-sensitive potassium channel (K_ATP_) opener, has been investigated as a pharmacological strategy to decrease mortality in cardiac surgery [8–10]. The intraoperative and postoperative use of levosimendan is mainly a rescue strategy for patients with difficult weaning from cardiopulmonary bypass (CPB) or in cases of LCOS. One recent meta-analysis showed that levosimendan seems to be the most effective drug in decreasing mortality after cardiac surgery [11], and another showed that the reduction in mortality and postoperative complications is driven by studies where levosimendan was used in patients with low LVEF [12].

Two randomized controlled trials (RCTs) published at the beginning of 2017 have not shown beneficial effects of the use of levosimendan compared with placebo in patients with preoperative severely depressed LVEF [13] or in a mixed population of patients with either preoperative severely depressed LVEF or profound intra-/postoperative cardiovascular dysfunction [14]. Nonetheless, researchers in the first study reported a beneficial effect in a subgroup of patients with a more severe reduction of LVEF [13]. Therefore, we aimed to conduct a new meta-analysis in light of the recently published RCTs, specifically examining the efficacy of levosimendan compared with placebo in decreasing mortality and need for renal replacement therapy (RRT) in high-risk cardiac surgery.

## Methods

### Eligibility criteria

We conducted a systematic search and meta-analysis of RCTs comparing levosimendan with placebo in high-risk patients undergoing cardiac surgery. We defined three subgroups of high-risk patients: (1) those with a preoperative severely depressed LVEF (<35%, the low LVEF subgroup), (2) patients with intra- and/or postoperative cardiovascular dysfunction requiring high pharmacological and/or mechanical support (LCOS subgroup), and (3) a mixed population of the previous two groups (low LVEF and LCOS subgroup). We excluded studies comparing levosimendan with other pharmacological strategies such as dobutamine and milrinone.

### Search strategy and criteria

Using the NHS Library Evidence tool, we undertook a systematic web-based advanced literature search of studies evaluating the use of levosimendan in high-risk cardiac surgery. We followed the approach suggested by the Preferred Reporting Items for Systematic Reviews and Meta-Analyses (PRISMA) statement for reporting systematic reviews and meta-analyses [15], and a PRISMA checklist is provided separately (Additional file 1). A computerized search of the MEDLINE (PubMed) and Embase databases was conducted from inception until March 30, 2017, to identify relevant articles. Our core search combined a group of findings containing the term “levosimendan” with a second group including the words “cardiac surgery” or “coronary artery bypass grafting” or “CABG.” Inclusion criteria were prespecified according to the PICOS (population, intervention, comparison, outcomes, and study design) approach (Table 1).

**Table 1:** PICOS approach for selecting clinical studies in the systematic search

PICOS	Criteria
1. Participants	High-risk patients undergoing cardiac surgery, defined by preoperative severely depressed LVEF (<35%) and/or intra-/postoperative LCOS
2. Intervention	Levosimendan
3. Comparison	Placebo
4. Outcomes	Primary outcomes: mortality at longest follow-up, need for RRT Secondary outcomes: myocardial injury, supraventricular arrhythmias, acute kidney injury (risk, injury, or failure according to RIFLE criteria), duration of mechanical ventilation, development of LCOS (only for studies on preoperative severely depressed LVEF patients), intensive care unit and hospital lengths of stay, adverse events, or hypotension during drug infusion
5. Study design	Randomized controlled trials

*Abbreviations: LVEF* Left ventricular ejection fraction, *LCOS* Low cardiac output syndrome, *RIFLE* Risk, injury, failure; loss, end-stage renal disease, *RRT* Renal replacement therapy, *PICOS* Population, intervention, comparison, outcomes, and study design

We excluded prospective and retrospective studies, case series, experimental animal studies, book chapters, reviews, editorials, and letters to the editor. Study selection for determining eligibility for inclusion in the systematic review and data extraction was performed independently by four reviewers (FS, JBK, CS, AA). Discordances were resolved by involving other authors. Language restrictions were applied, and only manuscripts published in English, French, Spanish, German, or Italian were included. A manual search was conducted independently by three authors (FS, JBK, CS) to explore the reference lists for the findings of the systematic search.

### Quality assessment

Methodological quality of included RCTs was evaluated using the Cochrane Collaboration tool, which incorporates the following domains: selection, performance, detection, attrition, reporting, and other potential sources of bias [16].

### Groups and endpoints

We primarily compared the efficacy of levosimendan with placebo with regard to survival at longest follow-up reported and the need for RRT. A subgroup analysis was performed according to the three types of high-risk populations included in the selected studies (*see* “Eligibility criteria” section above), and only when at least three studies were available in any of the subgroups. The following secondary endpoints were evaluated: the presence of myocardial injury; the incidence of atrial fibrillation (AF) and supraventricular arrhythmias; the occurrence of acute kidney injury (AKI) risk, injury, or failure (according to the RIFLE [risk, injury, failure; loss, end-stage renal disease] criteria); the duration of mechanical ventilation (MV); the intensive care unit (ICU) and hospital lengths of stay (LOS); the incidence of any adverse events; and the incidence of hypotension during drug infusion. Moreover, for the subgroup of studies including only patients with preoperative severely reduced LVEF, we evaluated the development of intra- and postoperative LCOS.

Two types of sensitivity analysis were initially planned. The first was conducted with a leave-one-out approach for the analyses including at least four studies. The second was planned by excluding the studies with a moderate and high risk of bias with the condition that at least four studies could be included. A third sensitivity analysis was added after the selection of findings. A three-arm RCT randomized patients to receive preoperative levosimendan alone vs the combination of levosimendan and intra-aortic balloon pump (IABP) vs IABP alone. The results from the first two arms were collected and included in this third sensitivity analysis because the only difference between such arms was the use of levosimendan [17].

### Statistical analysis

Analyses were conducted only for outcomes reported in a minimum of three studies. The Mantel-Haenszel method was used to analyze dichotomous outcomes, and results are reported as ORs with 95% CIs and two-tailed *p* values. Continuous outcome differences were analyzed using an inverse variance model with a 95% CI, and values are reported as standard mean difference (SMD). The *p* values were two-tailed. In both cases, *p* values were considered significant if < 0.05. The presence of statistical heterogeneity was assessed using the Cochran *Q* test. Heterogeneity was likely if *Q* > *df* suggested and was confirmed if *p* ≤ 0.10. Quantification of heterogeneity was performed, and *I* ^2^ values ranging from 0 to 24.9%, 25% to 49.9%, 50% to 74.9%, and > 75% were considered as none, low, moderate, and high heterogeneity, respectively. If heterogeneity was quantified as low or above, a random effects model was also used for sensitivity analyses [18].

## Results

Our systematic search identified a total of 601 findings via an NHS Library Evidence search. No other findings were retrieved manually. After removal of duplicates, 423 findings were screened and 171 were excluded because they were not focused on the topic of interest. As shown in the PRISMA flow diagram in Additional file 2, after the evaluation of the remaining 252 findings, only 6 RCTs were judged to be of interest for our quantitative analyses: 5 RCTs included only patients with preoperative severely depressed LVEF [13, 19–22], and 1 RCT included both patients with preoperative severely depressed LVEF and patients with intra-/postoperative LCOS [14]. No study was focused only on patients with intra-/postoperative LCOS. A list of the RCTs excluded despite enrolling patients with severely depressed LVEF (i.e., control subjects receiving milrinone or dobutamine) is provided separately (Additional file 3). Overall, data for up to 1728 patients were available, and data for up to 1224 patients were available for the subgroup of patients with preoperative severely reduced LVEF. Table 2 shows the characteristics of these studies, also including the timing of drug administration. Table 3 summarizes the results of primary and secondary outcome analyses.

**Table 2:** Population included in studies selected for meta-analysis

Author, study [reference]	Patients (*n*) and operations	LVEF cutoffs	Administration timing and dosages
Low LVEF only			
Erb et al., 2014 [19]	33 On-pump CABG (with or without valve)	<30%	Before incision 12.5-mg total dose at 0.1 μg∙kg^−1^∙minute^−1^
Levin et al., 2012 [20]	252 On-pump CABG only	<25%	Preoperative 10-μg/kg bolus; 0.1 μg∙kg^−1^∙minute^−1^ for 23 h
Mehta et al., 2017 [13]	849 On-pump cardiac surgery	<35%	Before incision 0.2 μg/kg/minute for 1 h; 0.1 μg∙kg^−1^∙minute^−1^ for 23 h
Shah et al., 2014 [21]	50 Off-pump CABG only	<30%	Preoperative 0.133 μg∙kg^−1^∙minute^−1^ for 24 h
Sharma et al., 2014 [22]	40 CABG and mitral valve repair	<30%	Preoperative 200 μg∙kg^−1^ for 24 h
Lomivorotov et al., 2012^a^ [17]	60 On-pump CABG only	<35%	Before incision 12-μg∙kg^−1^ bolus; 0.1 μg∙kg^−1^∙minute^−1^ for 24 h
LCOS only			
–	–	–	–
Low LVEF and LCOS			
Landoni et al., 2017 [14]	504 All cardiac surgery	<25%/or LCOS^b^	Mainly postoperative^b^ 0.05 μg∙kg^−1^∙minute^−1^ for 48 h or until ICU discharge

*Abbreviations: CABG* Coronary artery bypass grafting, *LCOS* Low cardiac output syndrome, *LVEF* Left ventricular ejection fraction

The studies are classified according to the subgroup of low LVEF and/or LCOS. We also report the number of patients in each study, the timing of levosimendan (placebo) administration, and the outcomes of interest of our meta-analysis reported by each study

^a^The study of Lomivorotov et al. [17] was a three-arm study with patients with low LVEF receiving preoperative levosimendan and intra-aortic balloon pump (IABP) vs levosimendan alone vs IABP alone. The data from the first two groups were included in a sensitivity analysis

^b^In this trial, only 4% of patients were randomized according to a preoperative low LVEF, 19% according to the need for IABP, 12% for difficult weaning from cardiopulmonary bypass, and 65% for postoperative LCOS

**Table 3:** Summary of main results of primary and secondary outcomes

					Heterogeneity	
Outcome analyzed	Studies	Patients	OR or SMD (95% CI)	*p* Value	*I* ^2^ Statistic	*p* Value
Mortality overall	6	1728	OR 0.64 (0.37, 1.11)	0.11	42%	0.12
**Mortality in low LVEF**	**5**	**1224**	**OR 0.51 (0.32, 0.82)**	**0.005**	**0%**	**0.66**
**Need for RRT overall**	**6**	**1728**	**OR 0.63 (0.42, 0.94)**	**0.02**	**0%**	**0.91**
**Need for RRT in low LVEF**	**5**	**1224**	**OR 0.55 (0.31, 0.97)**	**0.04**	**0%**	**0.90**
AF and SVT overall	5	1695	OR 0.62 (0.32, 1.18)	0.15	79%	0.0007
AF and SVT in low LVEF	4	1141	OR 0.52 (0.19, 1.40)	0.20	84%	0.0003
Myocardial damage overall	4	1645	OR 0.89 (0.52, 1.53)	0.68	29%	0.24
Myocardial damage in low LVEF	3	1141	OR 0.60 (0.15, 2.41)	0.47	53%	0.12
**Mechanical support overall**	**6**	**1728**	**OR 0.38 (0.13, 1.10)**	**0.07**	**82%**	**<0.0001**
**Mechanical support in low LVEF**	**5**	**1224**	**OR 0.29 (0.09, 1.00)**	**0.05**	**85%**	**<0.0001**
Hypotension overall	5	1695	OR 1.41 (0.92, 2.18)	0.12	0%	0.54
Hypotension in low LVEF	4	1191	OR 1.31 (0.82, 2.08)	0.26	0%	0.52
**LCOS in low LVEF**	**4**	**1191**	**OR 0.55 (0.38, 0.79)**	**0.001**	**9%**	**0.35**
**AKI (risk, injury, failure of RIFLE criteria)**	**3**	**817**	**OR 0.64 (0.44, 0.94)**	**0.02**	**9%**	**0.33**
Duration of MV overall	3	567	SMD −0.11 (−0.28, 0.05)	0.18	0%	0.61
**ICU LOS overall**	**4**	**1419**	**SMD −0.41 (−0.83, 0.02)**	**0.06**	**89%**	**<0.0001**
ICU LOS in low LVEF	3	962	SMD −0.78 (−1.90, 0.34)	0.17	92%	<0.0001
Hospital LOS overall	3	567	SMD −0.73 (−1.89, 0.43)	0.22	93%	<0.0001

*Abbreviations: AF* Atrial fibrillation, *AKI* Acute kidney injury, *ICU* Intensive care unit, *LCOS* Low cardiac output syndrome, *LOS* Length of stay, *LVEF* Left ventricular ejection fraction, *MV* Mechanical ventilation, *PICOS* Population, intervention, comparison, outcomes, and study design, *RIFLE* Risk, injury, failure; loss, end-stage renal disease, *RRT* Renal replacement therapy, *SMD* Standard mean difference, *SVT* Supraventricular tachycardia

Results are presented as OR or SMD, as appropriate, with 95% CI. Results presented in *bold* are statistically significant or with a trend toward statistically significant result

### Primary outcomes

**Fig. 1:**
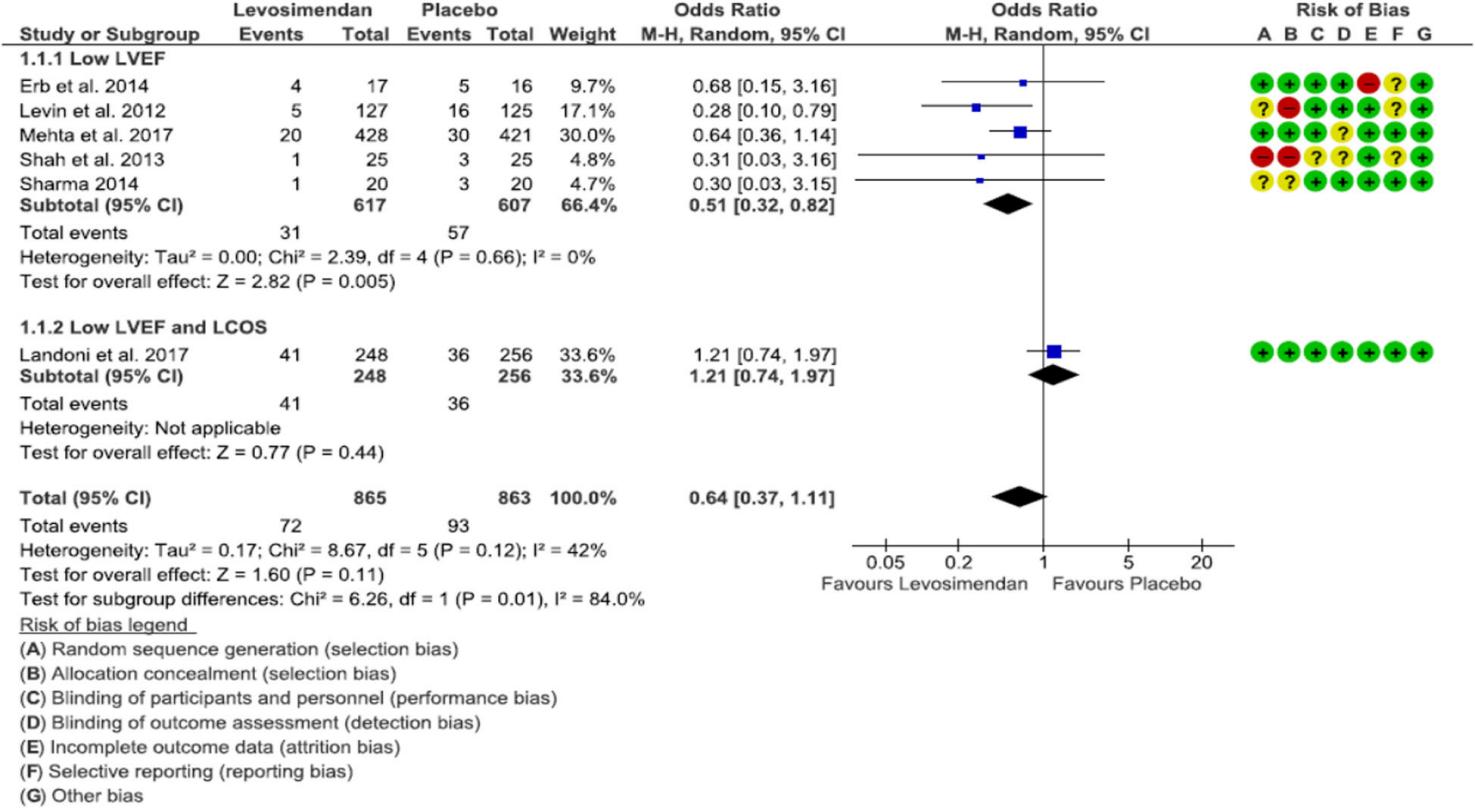
Forest plot depicting analysis of the risk of mortality at longest follow-up in patients treated with levosimendan vs placebo. *LVEF* Left ventricular ejection fraction, *LCOS* Low cardiac output syndrome, *M-H* Mantel-Haenszel

**Fig. 2:**
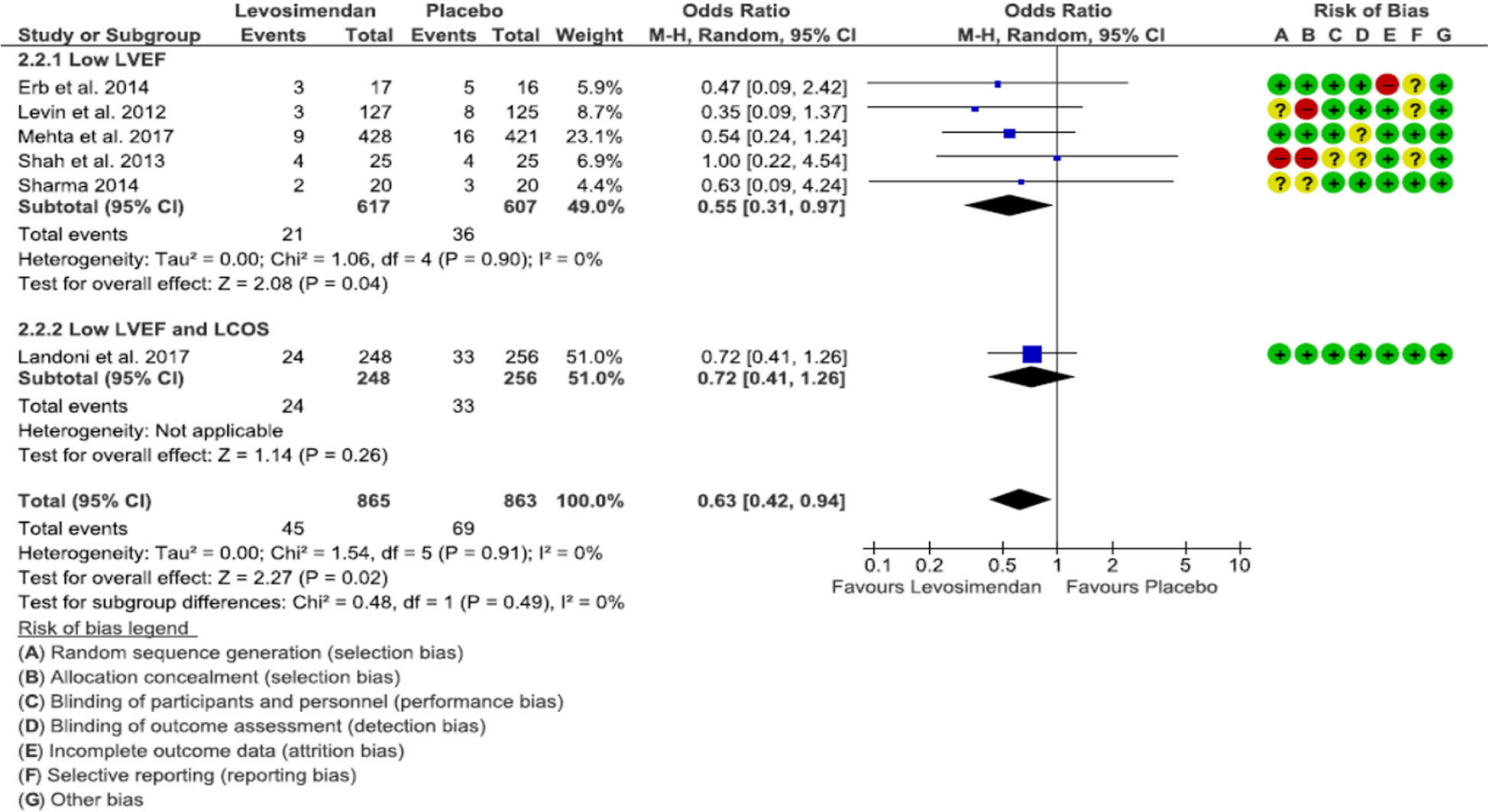
Forest plot depicting analysis of the risk for postoperative renal replacement therapy in patients treated with levosimendan vs placebo. *LVEF* Left ventricular ejection fraction, *LCOS* Low cardiac output syndrome, *M-H* Mantel-Haenszel

In six studies [13, 14, 19–22], both mortality and need for RRT were reported and were included in the primary outcome analysis. Mortality was similar overall (OR 0.64 [0.37, 1.11], *p* = 0.11, *I* ^2^ = 42%), but in the subgroup of patients with low LVEF, levosimendan showed a significantly lower mortality (OR 0.51 [0.32, 0.82], *p* = 0.005, *I* ^2^ = 0%) (Fig. 1). Need for RRT was significantly lower in the levosimendan group both overall (OR 0.63 [0.42, 0.94], *p* = 0.02, *I* ^2^ = 0%) and in the subgroup of patients with low LVEF (OR 0.55 [0.31, 0.97], *p* = 0.04, *I* ^2^ = 0%) (Fig. 2).

### Secondary outcomes

Researchers in five studies reported the incidence of postoperative AF and supraventricular arrhythmias, using different criteria (with a time frame of assessment not always reported) [13, 14, 20–22]. There was no difference in the incidence of postoperative AF and supraventricular arrhythmias between levosimendan and placebo, either overall (OR 0.62 [0.32, 1.18], *p* = 0.15, *I* ^2^ = 79%) or in the subgroup with low LVEF (OR 0.52 [0.19, 1.04], *p* = 0.20, *I* ^2^ = 84%).

Researchers in four studies reported the incidence of postoperative myocardial damage, using different criteria and time frames of assessment [13, 14, 20, 22]. There was no difference in the incidence of postoperative myocardial damage between levosimendan and placebo, either overall (OR 0.89 [0.52, 1.53], *p* = 0.68, *I* ^2^ = 29%) or in the subgroup with low LVEF (OR 0.60 [0.15, 2.41], *p* = 0.47, *I* ^2^ = 53%).

Researchers in six studies reported the need for postoperative cardiac mechanical support [13, 14, 19–22]. There was a trend toward a lower incidence of support with levosimendan, both overall (OR 0.38 [0.13, 1.10], *p* = 0.07, *I* ^2^ = 82%) and in the subgroup with low LVEF (OR 0.29 [0.09, 1.00], *p* = 0.05, *I* ^2^ = 85%).

The incidence of hypotension during drug infusion was reported in five studies [13, 14, 20–22]. There was no difference in the incidence of hypotension both overall (OR 1.41 [0.92, 2.18], *p* = 0.12, *I* ^2^ = 0%) and in the subgroup with low LVEF (OR 1.31 [0.82, 2.08], *p* = 0.26, *I* ^2^ = 0%).

Researchers in only three studies reported some data on AKI. In particular, Landoni et al. [14] reported the incidence of risk, injury, and failure according to the RIFLE classification, whereas investigators in other two studies [20, 21] reported the number of patients developing a serum creatinine increase at least > 50% from baseline (with or without oliguria), separating them from those requiring RRT. Such criteria equal at least the “risk” stage of the RIFLE classification. On the basis of pooling the results of these studies, levosimendan significantly reduced the risk of AKI compared with placebo (OR 0.64 [0.44, 0.94], *p* = 0.02, *I* ^2^ = 9%). Researchers in only three studies reported the duration of MV [14, 19], and they found no difference between levosimendan and placebo, either overall (SMD −0.11 [−0.28, 0.05], *p* = 0.18, *I* ^2^ = 0) or in the subgroup with low LVEF (SMD −0.19 [−0.66, 0.27], *p* = 0.41, *I* ^2^ = 0).

**Fig. 3:**
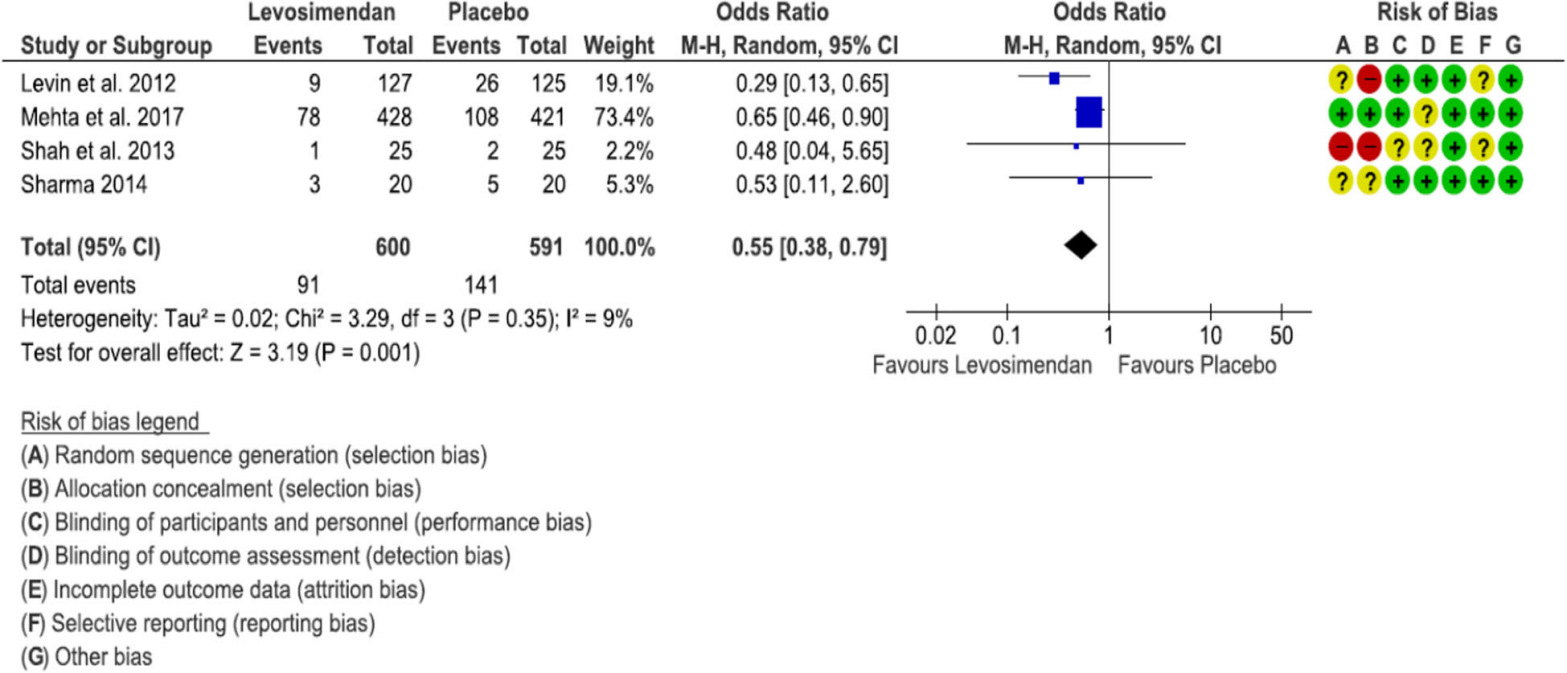
Forest plot depicting analysis of the risk for postoperative low cardiac output syndrome in patients treated with levosimendan vs placebo and with low preoperative left ventricular ejection fraction. *M-H* Mantel-Haenszel

The ICU LOS was reported by investigators in four studies [13, 14, 19, 22]. There was a trend toward a shorter overall ICU stay in the levosimendan group (SMD −0.41 [−0.83, 0.02], *p* = 0.06, *I* ^2^ = 89%), but not when the subgroup with low LVEF only was analyzed (SMD −0.78 [−1.90, 0.34], *p* = 0.17, *I* ^2^ = 92%) The hospital LOS was reported by researchers in three studies [14, 19, 22], and there were no differences between levosimendan and placebo (SMD −0.73 [−1.89, 0.43], *p* = 0.22, *I* ^2^ = 93%). Finally, researchers in four studies of the subgroup of RCTs including only patients with preoperative low LVEF reported the incidence of postoperative LCOS [13, 20, 21]. There was a significantly lower incidence of LCOS in patients treated with levosimendan (OR 0.55 [0.38, 0.79], *p* = 0.001, *I* ^2^ = 9%) (Fig. 3).

### Risk of bias assessment and sensitivity analyses

The two recent RCTs [13, 14] and one study were scored as having a low risk of bias [22], whereas the other three studies had at least a moderate risk of bias [19–21]. Because half of the RCTs were scored with at least moderate risk of bias, the sensitivity analysis including only RCTs at low risk (three studies) was not conducted [19–22]. The funnel plots of the two primary outcomes suggest no significant risk of publication bias.

We performed a series of sensitivity analyses with the leave-one-out approach for all the analyses including a minimum of four studies (thus remaining with at least three studies). With regard to mortality, the only change was the nonsignificant reduction in mortality with levosimendan in the subgroup with low LVEF only when removing the study by Levin et al. [20] (changed to *p* = 0.05). No other changes were seen by taking out any of the other five studies individually.

For postoperative RRT, exclusion of either the study by Mehta et al. [13] or the one by Levin et al. [20] demonstrated a trend toward overall lower need for RRT in the levosimendan group (*p* = 0.07 and *p* = 0.06, respectively). Moreover, in the subgroup with low LVEF only, exclusion of one of these studies [13, 20] made the difference between groups nonsignificant (*p* = 0.13 and *p* = 0.11, respectively). No changes were found after removal of any of the other four studies. Details of sensitivity analyses for secondary outcomes are provided separately (Additional file 4).

The third sensitivity analysis was conducted by adding the results of the study of Lomivorotov et al. [17], in which the authors randomized patients with preoperative ejection fraction < 35% to three arms: preoperative levosimendan alone vs combination of levosimendan and IABP vs IABP alone. The results from the first two arms were collected. This study provided data on most of the outcomes of interest of our meta-analysis, and its inclusion did not change any of the results for primary and secondary outcomes.

## Discussion

We conducted the present meta-analysis because of the discordance between the results of two recent large RCTs investigating the use of levosimendan in high-risk patients undergoing cardiac surgery [13, 14] and the results of previously published meta-analyses [11, 12]. The two recent RCTs failed to show a beneficial effect of levosimendan compared with placebo [13, 14], whereas the two meta-analyses had opposite results [11, 12]. Another reason for conducting our study was the evidence in one RCT of a beneficial effect of levosimendan in the subgroup of patients with more severe reduction of LVEF (<25%) and in those undergoing isolated coronary artery bypass grafting (CABG); moreover, such RCTs showed a trend toward lower mortality at 90 days for levosimendan (4.7% vs placebo 7.1%, *p* = 0.12) [13].

Although the two recent RCTs included a broader spectrum of cardiac surgical interventions and were not focused exclusively on patients undergoing CABG [13, 14], previous studies were focused mostly on patients with coronary artery disease undergoing CABG (isolated [20, 21] or CABG with or without valve surgery [19, 22]). Moreover, such studies have used a more restrictive cutoff for preoperative LVEF (<25% [20] or < 30% [19, 21, 22]).

The primary endpoint of our meta-analysis was the difference in mortality and need for postoperative RRT in high-risk patients undergoing cardiac surgery receiving levosimendan compared with placebo. We therefore excluded studies comparing levosimendan with other pharmacological strategies, such as dobutamine and milrinone. We found only six RCTs comprising over 1700 patients, 1200 of whom over belonged to the subgroup of patients with preoperative severely reduced LVEF only (<35%). We investigated mortality and the need for postoperative RRT as primary outcomes. Whereas overall mortality was not different, levosimendan compared with placebo significantly reduced mortality in the subgroup of patients with preoperative severely reduced LVEF. Moreover, levosimendan significantly decreased the need for postoperative RRT after cardiac surgery, both overall and in the subgroup of patients with preoperative severely reduced LVEF (<35%).

These findings are not surprising, because even the larger RCT [13] showed a trend toward beneficial effects of levosimendan in patients with more depressed LVEF (<25%) and also among those with more advanced chronic kidney disease (estimated glomerular filtration rate < 60 ml/minute). Several aspects, however, should be taken into account. Levosimendan is a calcium-sensitizing inotrope as well as a K_ATP_ opener, and it has organ-protective properties. The opening of K_ATP_ channels located in the plasma membrane of vascular smooth muscle cells and at the level of mitochondria improves energy homeostasis, protecting the heart from calcium overload and oxidative injury [23, 24] and optimizing mitochondrial energy balance [24]. At coronary artery levels, levosimendan produces vasodilation through the increase in extracellular potassium, resulting in an increase in blood flow to ischemic myocardial regions [25]. This ultimately results in improved myocardial function, as shown by enhanced arterial-ventricular coupling [26]. For such reasons, patients with coronary artery disease undergoing CABG may experience a greater benefit from levosimendan by additive protection in areas at high risk of perioperative myocardial injury. Therefore, it is likely that our results differ from those of the recent RCTs, because our meta-analysis also included four studies that randomized only patients undergoing CABG (with or without valve surgery). A favorable effect of levosimendan might have been blunted in the larger RCTs that included a wider spectrum of patients undergoing cardiac surgery.

The advantageous renal effects of levosimendan are reinforced by a lower number of patients with postoperative AKI according to the RIFLE criteria. We believe that such results can be attributed primarily to an improvement in systemic hemodynamics. The increase in cardiac output and the reduction in right-sided pressures, including central venous pressure [27], may decrease renal vein pressure. Such effects may increase renal flow per se. Additionally, because renal blood flow depends on vascular resistance, the local vasodilation obtained by levosimendan through the K_ATP_ channels in the afferent arterioles increases renal perfusion, thus increasing glomerular filtration pressure and filtration rate. Such effects on renal arterioles are different from those of other inotropes/vasoconstrictor agents. Also, nonhemodynamic effects such as preconditioning, anti-inflammatory effects, and antiapoptotic properties may have played a role in improving renal function [28].

The preoperative use of levosimendan has also been studied in CABG patients with preserved LVEF, and, although a reduction in myocardial injury and an increase in cardiac index in the levosimendan group were shown, the study failed to show a clear benefit of this strategy [29]. In the studies included in our meta-analysis, levosimendan has been used with varying timing of administration. In three studies, levosimendan has been given preoperatively 24 h before surgery [20–22], whereas researchers in two studies initiated drug administration just before surgery [13, 19], and another study included mainly patients after difficult weaning from CPB or patients who developed profound cardiovascular dysfunction in the ICU [14].

It is interesting to note that levosimendan was associated with a lower incidence of LCOS in the subgroup of patients with low LVEF only and with a trend toward lower use of mechanical circulatory support, both overall and in the subgroup of patients with low LVEF only. In our study, we did not perform a comparison between levosimendan and other inotropes. Levosimendan has a mechanism different from that of other inotropes, which typically increase intracellular calcium levels with subsequent higher myocardial oxygen demand, adverse effects [30], and higher mortality [31, 32]. The rise of intracellular calcium levels does not happen with levosimendan, and therefore the drug does not increase in myocardial oxygen consumption [33].

One of the main issues with the use of levosimendan is related to its pharmacological properties. Beneficial ischemic myocardial preconditioning may be obtained if administration of the drug is performed several hours before the insult [9], but, as reported above, researchers in only three studies admitted patients to the ICU 24 h before surgery in order to start the drug infusion [20–22]. In two other studies, investigators started the administration of levosimendan after anesthesia induction without a bolus [13, 19], and in one study, researchers used levosimendan mainly for difficult weaning from CPB or for postoperative LCOS [14]. It should be noted that when levosimendan is used at the beginning of surgery, it may be useful to administer a drug bolus to reach a peak concentration that is then maintained by infusion [9]. Therefore, in these studies, the beneficial effects of levosimendan may have been reduced by the late administration, the absence of a bolus, and possibly a dilution through the CPB circuit. It should be noted that bolus administration increases the risk of hypotension requiring the administration of noradrenaline, especially when given in a high dose (24 μg/kg) [34]; however, the experts of the European consensus on the use of perioperative levosimendan do not recommend a bolus, and in cases where a bolus is administered, they mostly suggest lower dosages (6 μg/kg, 67%; 12 μg/kg, 29%; 24 μg/kg, 4%) [9].

Another fundamental aspect in the cardiac surgery patient is the presence of diastolic dysfunction associated with depression of contractility. In these cases, levosimendan has shown improvements [35] or neutral effects on diastolic function [36], which instead is worsened by the use of catecholamines [30] and unchanged by inhibitors of phosphodiesterase [37, 38]. A large number of patients undergoing cardiac surgery are currently treated with beta-blockers, and levosimendan can offer advantages because it does not interfere with the receptor-mediated mechanisms of catecholamines and does not impair left ventricular diastolic function [39, 40]. Most of the patients with heart failure and/or severe systolic dysfunction are treated with beta-blockers [41, 42]. It is worth noting that, apart from two small studies (Shah et al. [21] and Sharma et al. [22], where a minority of patients were receiving beta-blockade therapy [14% and 12.5%, respectively]), most of the patients in the other studies of our meta-analysis were treated with beta-blockade therapy (Mehta et al. [13], ~ 80%; Landoni et al., [14], > 60%; Levin et al. [20], ~ 85%; Erb et al. [19], > 90%). Whether patients with preoperative treatment with beta-blockers may benefit more from levosimendan remains speculative and should be explored in subgroup analyses. Furthermore, levosimendan could offer advantages in patients with right ventricular systolic and diastolic dysfunction, with or without associated pulmonary hypertension [43–45].

### Limitations

Our results should be interpreted cautiously because we found a reduced number of studies, and three of them [19–21] had moderate risk of bias. Moreover, we describe the clinical heterogeneity of the included studies with regard to the timing of levosimendan administration and the surgical population. Three studies administered levosimendan 24 h before surgery [20–22], but this may not always be feasible, especially in patients with urgent need for surgery.

Our results partially contradict those of the two recent RCTs [13, 14], but it should be kept in mind that such studies also have several limitations. In the CHEETAH trial [14], most of the patients (96%) received levosimendan after CPB or postoperatively for profound cardiovascular dysfunction (LCOS). On one hand, a low-dose infusion of levosimendan was used, which was possibly explained by severe clinical conditions (hypotension and/or high doses of vasoactive drugs), but on the other hand, such a dose may not allow full achievement of the intended pharmacological effect. There was almost 13% mortality in both groups, and this was almost double the mortality expected by the initial sample size calculation. This mortality could be explained by the high doses of inotropes, which are independently associated with mortality in cardiac surgery [31, 32]. The other recent LEVO-CTS study [13] was a phase III trial designed for Food and Drug Administration approval of levosimendan. It was a well-designed RCT with contributions from European colleagues with a high level of experience and involved 70 centers over 26 months. Thus, on average, there was not a large use of the drug per center, introducing a potential bias in the results. Nonetheless, the same study showed a trend toward lower mortality at 90 days for levosimendan (*p* = 0.12).

## Conclusions

Levosimendan reduces mortality in patients with preoperative severely reduced ejection fraction (<35%). Moreover, levosimendan significantly reduces the need for RRT after high-risk cardiac surgery.

## Additional files

Additional file 1: PRISMA Checklist. (DOC 63 kb)

Additional file 2: PRISMA Flowchart. (DOC 32 kb)

Additional file 3: Reasons for exclusion of randomized controlled trials including patients with left ventricular ejection fraction ≤35%. Studies are divided in peer-reviewed publications and conference abstracts. *RRT* renal replacement therapy. (DOCX 14 kb)

Additional file 4: Sensitivity analyses of secondary outcomes with “leaving one out at time” approach. (DOCX 14 kb)

## Abbreviations

AF: Atrial fibrillation
AKI: Acute kidney injury
CABG: Coronary artery bypass grafting
CPB: Cardiopulmonary bypass
IABP: Intra-aortic balloon pump
ICU: Intensive care unit
K_ATP_: ATP-sensitive potassium channel
LCOS: Low cardiac output syndrome
LOS: Length of stay
LVEF: Left ventricular ejection fraction
M-H: Mantel-Haenszel
MV: Mechanical ventilation
PICOS: Population, intervention, comparison, outcomes, and study design
PRISMA: Preferred Reporting Items for Systematic Reviews and Meta-Analyses
RCT: Randomized controlled trial
RIFLE: Risk, injury, failure
loss: end-stage renal disease
RRT: Renal replacement therapy
SMD: Standard mean difference
SVT: Supraventricular tachycardia
