# Supplementary material for: Levosimendan for patients with severely reduced left ventricular systolic function and/or low cardiac output syndrome undergoing cardiac surgery: a systematic review and meta-analysis
Source: Crit Care. 2017 Oct 19;21:252. doi: 10.1186/s13054-017-1849-0 (PMC5648477; doi:10.1186/s13054-017-1849-0)
Supplement: Supplementary file 2 — PRISMA Flowchart. (DOC 32 kb) [file 13054_2017_1849_MOESM2_ESM.doc]

**Screening**

**Included**

**Eligibility**

**Identification**

Records identified through EMBASE, MEDLINE (Pubmed)
(n=601)

Additional records identified through other sources
(n=0)

Records after duplicates removed
(n=423)

Records screened
(n=423)

Records excluded
(n=171)

Full-text articles assessed for eligibility
(n=252)

Full-text articles excluded, with reasons:

Animal studies (n=36)

Reviews (n=75)

Letters (n=18)

Case studies (n=27)

Retrospective (n=28)

Non-RCTs (n=20)

RCTs LVEF>35% (n=32)

RCTs LVEF≤35% but not otherwise qualifying (n=9)

Studies included in qualitative synthesis
(n=7)

Studies included in quantitative synthesis (meta-analysis)
(n=6)

**SUPPLEMENTAL DIGITAL CONTENT 2**

PRISMA Flowchart of study selection. LVEF: left ventricular ejection fraction; RCT: randomized controlled trial.
