# Supplementary material for: Levosimendan for patients with severely reduced left ventricular systolic function and/or low cardiac output syndrome undergoing cardiac surgery: a systematic review and meta-analysis
Source: Crit Care. 2017 Oct 19;21:252. doi: 10.1186/s13054-017-1849-0 (PMC5648477; doi:10.1186/s13054-017-1849-0)
Supplement: Supplementary file 4 — Sensitivity analyses of secondary outcomes with “leaving one out at time” approach. (DOCX 14 kb) [file 13054_2017_1849_MOESM4_ESM.docx]

**SUPPLEMENTAL DIGITAL CONTENT 4**

Sensitivity analyses of secondary outcomes with “leaving one out at time” approach.

The exclusion of Mehta et al.[^13^](file:///C:\Users\fgsanfilippo\Desktop\LCO%20SYNDROME%20TALK\Meta-analysis\version%2010%20Levo%20vs%20%20placebo.docx#_ENREF_13) changed the risk of postoperative AF and/or SVT with significantly lower incidence in patients treated with levosimendan both overall (p=0.04) and in the subgroup with low LVEF only (p=0.002). No changes made by removal of any of the other four studies. With respect to mechanical support, the exclusion of Mehta et al.[^13^](file:///C:\Users\fgsanfilippo\Desktop\LCO%20SYNDROME%20TALK\Meta-analysis\version%2010%20Levo%20vs%20%20placebo.docx#_ENREF_13) changed results into a lower use of mechanical support in the levosimendan group both overall (p=0.003) and in low LVEF subgroup (p<0.00001). No changes made by removal of any of the other five studies. There were no changes in hypotension by removing any of the five studies. Similarly, no differences were seen in the sensitivity analyses for ICU-LOS (four studies) and for postoperative LCOS (four studies).
