# Supplementary material for: Levosimendan for patients with severely reduced left ventricular systolic function and/or low cardiac output syndrome undergoing cardiac surgery: a systematic review and meta-analysis
Source: Crit Care. 2017 Oct 19;21:252. doi: 10.1186/s13054-017-1849-0 (PMC5648477; doi:10.1186/s13054-017-1849-0)
Supplement: Supplementary file 3 — Reasons for exclusion of randomized controlled trials including patients with left ventricular ejection fraction ≤35%. Studies are divided in peer-reviewed publications and conference abstracts. RRT renal replacement therapy. (DOCX 14 kb) [file 13054_2017_1849_MOESM3_ESM.docx]

**SUPPLEMENTAL DIGITAL CONTENT 3**

Reasons for exclusion of randomized controlled trials including patients with left ventricular ejection fraction ≤35%. Studies are divided in peer-reviewed publications and conference abstracts. RRT= renal replacement therapy.

| Author | Year | Reason for Exclusion |
| --- | --- | --- |
| *Peer-reviewed publications* |  |  |
| Abacilar et al. | 2013 | No specified ejection fraction criteria; mortality and RRT outcomes not reported |
| Al Shawaf et al. | 2006 | Milrinone used as control |
| Alvarez et al. | 2006 | Dobutamine used as control |
| Alvarez et al. | 2013 | Dobutamine used as control |
| De Hert et al. | 2008 | Milrinone used as control |
| De Hert et al. | 2007 | Milrinone used as control |
| Lomivorotov et al. | 2011 | Intra-aortic balloon pump used as control |
| Sahin et al. | 2014 | Dopamine and/or dobutamine used as control |
| Temizturk et al. | 2016 | Levosimendan given in combination with sodium nitroprusside |
| *Conference abstracts* |  |  |
| Karabacak et al. | 2012 | Post-operative mortality outcomes not reported |
| Lomivorotov et al. | 2010 | Intra-aortic balloon pump used as control |
| Lomivorotov et al. | 2011 | Intra-aortic balloon pump used as control |
| Lomivorotov et al. | 2012 | Results reported in Lovimortov study discussed in manuscript |
| Lomivorotov et al. | 2013 | Intra-aortic balloon pump used as control |
| Pasyuga et al. | 2014 | No placebo group; three different strategies for levosimendan administration |
| Tzimas et al. | 2009 | Post-operative mortality and RRT outcomes not reported. |
